# Assessing Acceptability of COVID-19 Vaccine Booster Dose among Adult Americans: A Cross-Sectional Study

**DOI:** 10.3390/vaccines9121424

**Published:** 2021-12-02

**Authors:** Tesfaye Yadete, Kavita Batra, Dale M. Netski, Sabrina Antonio, Michael J. Patros, Johan C. Bester

**Affiliations:** 1 Kirk Kerkorian School of Medicine, University of Nevada, Las Vegas, NV 89102, USA; yadete@unlv.nevada.edu (T.Y.); antons1@unlv.nevada.edu (S.A.); PATROM1@unlv.nevada.edu (M.J.P.); 2 Office of Research, Kirk Kerkorian School of Medicine, University of Nevada, Las Vegas, NV 89102, USA; 3 Department of Medical Education and Office of Academic Affairs, Kirk Kerkorian School of Medicine, University of Nevada, Las Vegas, NV 89102, USA; Dale.netski@unlv.edu (D.M.N.); johan.bester@unlv.edu (J.C.B.)

**Keywords:** COVID-19, vaccine hesitancy, vaccine literacy, functional literacy, communicative literacy, critical literacy, vaccine confidence index, herd immunity, vaccine booster

## Abstract

Given the emergence of breakthrough infections, new variants, and concerns of waning immunity from the primary COVID-19 vaccines, booster shots emerged as a viable option to shore-up protection against COVID-19. Following the recent authorization of vaccine boosters among vulnerable Americans, this study aims to assess COVID-19 vaccine booster hesitancy and its associated factors in a nationally representative sample. A web-based 48-item psychometric valid survey was used to measure vaccine literacy, vaccine confidence, trust, and general attitudes towards vaccines. Data were analyzed through Chi-square (with a post hoc contingency table analysis) and independent-sample *t*-/Welch tests. Among 2138 participants, nearly 62% intended to take booster doses and the remaining were COVID-19 vaccine booster hesitant. The vaccine-booster-hesitant group was more likely to be unvaccinated (62.6% vs. 12.9%) and did not intend to have their children vaccinated (86.1% vs. 27.5%) compared to their non-hesitant counterparts. A significantly higher proportion of booster dose hesitant individuals had very little to no trust in the COVID-19 vaccine information given by public health/government agencies (55% vs. 12%) compared to non-hesitant ones. The mean scores of vaccine confidence index and vaccine literacy were lower among the hesitant group compared to the non-hesitant group. Compared to the non-hesitant group, vaccine hesitant participants were single or never married (41.8% vs. 28.7%), less educated, and living in a southern region of the nation (40.9% vs. 33.3%). These findings underscore the need of developing effective communication strategies emphasizing vaccine science in ways that are accessible to individuals with lower levels of education and vaccine literacy to increase vaccination uptake.

## 1. Introduction

The coronavirus-19 disease (COVID-19) has caused significant mortality and morbidity worldwide [1,2,3]. Vaccines against COVID-19 were developed by early 2021 and demonstrated outstanding protection against COVID-19 infections, hospitalizations, and deaths [4,5,6]. There has been a considerable discussion about the eventual need for a booster dose of COVID-19 vaccines, primarily in response to concerns about possible waning immunity, the transmission of breakthrough infections, and the emergence of new viral variants with increased transmissibility [7,8,9,10].

While protection against hospitalizations and deaths remained robust, several studies appeared to show a decline in protection against COVID-19 infections a few months after the initial vaccination. In a retrospective study in Israel, rates of breakthrough infections amongst fully vaccinated patients with the Pfizer-BioNTech vaccine were considerably higher among those vaccinated earlier than those who were immunized later [11]. Analysis of an observational study among fully vaccinated nursing home residents revealed that the vaccine efficacy dropped from 74.7% to only 53.1% in a few months [12]. Another study found that vaccines’ effectiveness against infections amongst New Yorkers waned from 91.7% to 79.8% [13]. However, this study also found that the vaccine remains highly effective against hospitalizations. Other studies reported a decline in vaccine effectiveness over time and increased potential for hospitalizations following breakthrough infections among immunocompromised patients [12,14,15,16,17]. In addition, a study performed in Israel demonstrated that administering a booster dose five months after completing the initial two-dose regimen increased protection against infection by 11.3-fold and severe illness by 19.5-fold [18]. Due to the potential for waning immunity and the emergence of new virus variants, there have been calls for the introduction of booster shots. Some argue that booster shots are not presently needed in the general population but concede that they may ultimately be needed because of these considerations [19].

Many countries have already started administering booster doses in the wake of breakthrough infections, arrivals of new variants, and a decline in long-term protection. As of 20 September 2021 (at the time of writing this manuscript), over twenty-one million booster doses have been given across the world [20]. Israel has been administering booster shots since 30 July 2021 [18,21]. In the United States of America (U.S.A.), the Center for Disease Control and Prevention (CDC) recommends that immunocompromised patients receive a booster shot [15]. Furthermore, the U.S. Food and Drug Administration (FDA) Vaccines and Related Biological Advisory Committee voted unanimously on 17 September 2021 to recommend a booster dose for individuals 65 years of age and older and individuals at high risk of severe COVID-19 [22]. Similarly, the United Kingdom (UK) is also planning to administer boosters in the autumn of 2021 [23,24].

The use of booster doses will at some point play an essential role in the public health response to the pandemic. However, the public’s acceptance of a booster dose emerges as a potential concern. As COVID-19 vaccination efforts have been hampered by hesitancy and mistrust [25,26,27], COVID-19 booster shot initiatives will likely face similar challenges. In order to design interventions and increase the uptake of the booster dose, it is imperative to quantify the baseline acceptance levels towards the booster dose. Therefore, this study aims to investigate acceptance for future booster doses and its associated factors among vaccine-eligible Americans.

## 2. Methods

### 2.1. Study Design, Setting, and Sampling

Data for this cross-sectional, descriptive, and exploratory study were collected from 14 July to 19 July 2021, using a commercial data collection and administration agency, Qualtrics, to recruit a nationally representative sample by gender, race, ethnicity, and regional distribution. Qualtrics is a marketing research company which utilizes its high-quality research panels and quota sampling to recruit specific target population groups. Additional details about the sampling strategy can be accessed at https://www.qualtrics.com/research-services/online-sample/ (accessed on 22 September 2021). Miller and colleagues have also described Qualtrics’ recruitment process for their commercial research panels and the advantages of online research panels and quota sampling techniques over traditional sampling methods [28]. Qualtrics recruits a panel of participants through a convenience sampling of sources acquired from partnerships with over 20 online sample providers. This recruitment method enables Qualtrics to provide researchers with diverse datasets that represent the population under study. The respondents are first randomly selected by the sample partners through traditional, actively managed, double-opt-in market panels that allow selecting participants that are likely to qualify. Social media can also be used to amass respondents when necessary.

### 2.2. Study Population and Selection Criteria

The study samples were obtained from currently available pools of research participants, who have consented to be communicated for future studies. To avoid heavy dependence on a single segment of the population, Qualtrics pooled samples from different sources across the nation. The recruitment of respondents was performed in an enforced quota sampling fashion to create a pool of participants representing the current U.S. demography. The enforced quota constraints closely matched the U.S. Census, as shown below in the Table 1.

Current U.S. residents aged at least 18 years with the ability to provide voluntary informed consent and understand English were eligible to participate in this study. Participants who did not meet these criteria were excluded from the study. Screening questions were added at the start of the survey to ensure adherence to the study’s eligibility criteria. In order to minimize self-selection and response bias, details of the actual survey were not shown until the participants passed the screening criteria. Survey respondents were compensated for their time through incentives, which may include cash, airline miles, charitable donations, sweepstakes entrance, vouchers, or gift certificates.

### 2.3. Ethical Considerations

The Institutional Review Board (IRB) granted this study a category two exemption (protocol # 1762717-2). Participants were informed of the significance and the objectives of the study. Participation in the survey was voluntary. No personal identifiers were acquired.

### 2.4. Quality Control and Data Privacy

Multiple data quality features were used in the survey to ensure data integrity and unique responses from each study participant. Digital fingerprinting and preventing ballot box stuffing restricted participants from answering the survey more than once. Participants were excluded if they completed the survey significantly quicker than other survey respondents due to the high possibility of a lack of thoughtfulness in their responses. Qualtrics and the research team members abided by all data privacy laws and regulations. The Qualtrics database does not store any confidential information of the respondents or panelists. Deidentified data were provided to the researchers in an excel sheet safeguarded on a password-protected device in a locked office. Access to these files was restricted to research team members.

### 2.5. Survey Instrument

This study utilized a 48-item web-based survey consisting of several psychometrically valid and reliable tools to measure the following constructs: attitudes towards other vaccines in general (9 items), confidence towards COVID-19 booster dose (8 items), vaccine literacy (14 items), and demographic questions (17 items). Questions related to general attitudes towards vaccines were adapted from prior studies that assessed vaccine hesitancy using a standardized tool to measure vaccine attitude [29,30]. To measure vaccine literacy, a self-reported questionnaire was used, originally based on the Ishikawa test for chronic non-communicable diseases [31]. The vaccine confidence index (VCI) was modified to apply to the COVID-19 vaccine specifically. This VCI was previously used in studies analyzing choice architecture for the influenza vaccine and in vaccine confidence projects in many countries [30,32,33].

### 2.6. Sampling and Sample Size

Participation and completion rates are difficult to calculate because respondents were sourced via various methods (e.g., email, online/mobile games, dashboard where they self-select into the survey, apps, etc.), making these values difficult to pinpoint. Qualtrics aggregates many online panel resources, and most use what are called “dynamic surveys” that are distributed in a dashboard style, where respondents see a dashboard of surveys that they likely qualify for. This can also include app-based recruitment, and a multitude of other methods. Some people may receive email notifications, but this is an older system of distributing surveys that is not widely used. The tracking of the respondents starts as soon as they choose to engage with the survey specifically; however, there is no specific survey invite that everyone sees across the project and, therefore, exactly how many invites are sent out cannot be reported. Out of the total survey entrants, screening criteria, speeding checks, quality control, and quota constraints are applied by Qualtrics to ensure delivery of high-quality and complete data to abide by the contractual agreement made between researchers and Qualtrics. Out of the total survey entrants, screening criteria, speeding checks, quality control, quota constraints are applied by Qualtrics to ensure the delivery of high-quality and complete data to abide by the contractual agreement made between researchers and Qualtrics. There was a total of 2138 complete entries in the dataset provided by Qualtrics and missing data analysis was not warranted. The Checklist for Reporting Results of Internet E-Surveys (CHERRIES) guidelines was followed to provide a thorough description of this web-based survey’s validity [34].

### 2.7. Sample Size Justification

G*Power software was used to conduct power analyses [34,35] to determine whether the sample was sufficient to detect the hypothesized effects. Using Cohen’s benchmarks of small effect sizes related to each statistical test (0.1 for Chi-square and 0.2 for *t*-test), alpha level 5%, and 95% power, the maximum sample size required was 1979. After factoring in 10% missing data, the estimated sample size was 2177, comparable to the current sample size utilized in this study.

### 2.8. Data Analyses

All analyses were conducted using IBM SPSS v.26 (IBM Corp, Armonk, NY, USA) and SAS 9.4. statistical software. Descriptive statistics were performed to compute the mean, standard deviations, counts, and proportions. Exploratory analyses were utilized to investigate the data distribution and assumptions. A Chi-square test with a post hoc contingency analysis was conducted to compare categorical variables among booster hesitant and non-hesitant groups by Chi-square test. The observed *p*-values were Bonferroni-corrected in multiple comparisons [36]. Continuous variables, such as the vaccine confidence, attitudes, and vaccine literacy, were compared using independent-sample *t*-tests or Welch’s *t*-test (wherever appropriate). Levene’s test for equality of variances was utilized to check the assumption of homogeneity of variances. A bootstrapping technique was also used to validate the statistical significance among groups. Effect sizes were computed to measure the strength of the effect that emerged from the sampled data. A subgroup analysis to compare vaccine literacy scores among hesitant and non-hesitant participants who have had “a lot” of trust in COVID-19 vaccine information was also conducted. A Checklist for statistical Assessment of Medical Papers (the CHAMP statement) was used for statistical reporting [37]. A multivariate logistic regression model was fit to generate an adjusted odds ratio. Estimates for the parameters were obtained through the maximum likelihood estimation method with 95% Wald’s confidence limits for the logistic model. The final model was selected based upon the Akaike information criterion (AIC) and the Schwarz criterion (SC) [38]. The significance level was set at 5%.

## 3. Results

**Figure A1:**
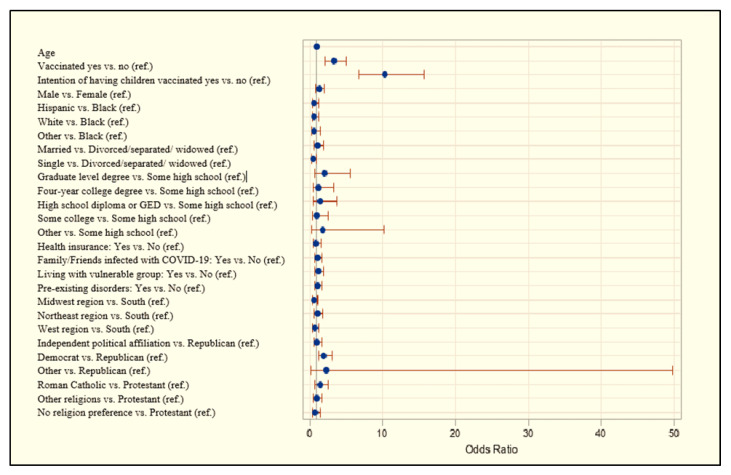
Forest plot indicating odd ratio estimates and Wald confidence intervals.

Out of 2138 total respondents, 1322 (61.8%, 95% CI: 59.7–63.9%) were willing to take the booster dose, and the remaining 38.2% (95% CI: 36.1–40.3%) were booster dose hesitant (Table 2). In comparison to those willing to take a booster dose, members of the booster dose hesitant group were relatively younger (42.5 years vs. 47.6 years), unvaccinated (62.6%, 95% CI: 59.2–65.9% vs. 12.9%, 95% CI: 11.1–14.8%; *p* < 0.001), and did not intend to have their children vaccinated (86.1%, 95% CI: 82.4–89.2% vs. 27.5%, 95% CI: 23.8–31.4%; *p* < 0.001). As shown in Table 2, 55.4% (95% CI: 51.9–58.8%) of females were in the hesitant group, compared to 46.2% (95% CI: 43.5–48.9%) in the non-hesitant group (*p* < 0.001), and the effect size, Cramer’s V, was small. Political affiliation also plays a significant role in determining whether one will get a booster dose as nearly 33% (95% CI: 29.5–36.0%) of Republicans were in the hesitant group, compared to 23% (95% CI: 20.9–25.5%) in the non-hesitant group (*p* < 0.001). Religiously unaffiliated participants were significantly more vaccine-hesitant than non-hesitant (35.5%, 95% CI: 32.3–38.9% vs. 27.4%, 95% CI: 24.9–29.8%; *p* < 0.001). As compared to the vaccine non-hesitant group, vaccine hesitant participants were single or never married (41.8%, 95% CI: 38.4–45.3% vs. 28.7%, 95% CI: 26.3–31.3%), uninsured (16.4%, 95% CI: 13.5–18.6% vs. 7.0%, 95% CI: 5.6–8.5%), less educated, and living in a southern region of the nation (40.9%, 95% CI: 37.5–44.4% vs. 33.3%, 95% CI: 30.7–35.9%; *p* < 0.001, Table 2). Living with a vulnerable family member, having COVID-19 positive friends or family members, and having pre-existing conditions were also significantly associated with booster dose acceptability. However, the effect size was small, ranging from 0.10 to 0.15 (Table 2). The results of multivariate logistic regression analysis indicated that acceptability towards primary series of COVID-19 vaccination, parents’ willingness to have their children vaccinated, and political affiliation were significant predictors of booster dose acceptability (Appendix A Figure A1). After controlling for all confounders, participants who had already received a primary dose of COVID-19 vaccine were more than thrice likely to accept booster doses (OR 3.32, 95% CI: 2.20–5.01; Appendix A Figure A1). Parents who were willing to have their children vaccinated had significantly higher odds of booster dose acceptability (OR 10.3; 95% CI: 6.78–15.77). Among all demographic variables, political affiliation was a significant predictor of booster dose acceptability. Being a democrat increased the likelihood of booster dose acceptability by nearly twice (OR 1.90, 95% CI: 1.17–3.10).

**Figure 1:**
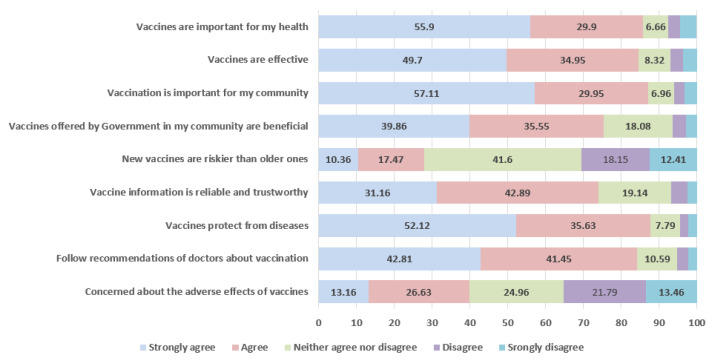
General attitudes towards vaccines among booster dose non-hesitant group.

**Figure 2:**
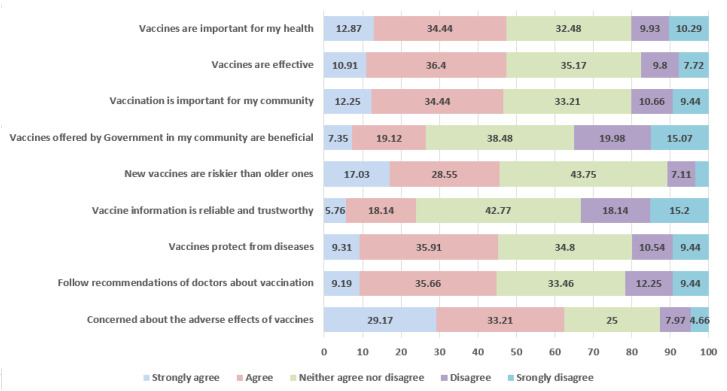
General attitudes towards vaccines among booster dose hesitant group.

As shown in Table 3, nearly 55% (95% CI: 51.7–58.7%) of booster-hesitant participants reported having no or very little trust in COVID-19 vaccine information compared to 15% (95% CI: 13.5–17.4%) in the non-hesitant group (*p* < 0.001), and the effect size, Cramer’s V, was moderately large [35]. Among participants willing to take the booster dose, attitudes towards vaccines, in general, were favorable (Figure 1 and Figure 2). When evaluating general attitudes towards vaccines, booster non-hesitant participants responded favorably to the statement “vaccines are important for my health” compared to booster-hesitant participants (85.8% vs. 47.31%). To the statement “vaccines are effective,” nearly 85% of individuals willing to take the booster shot either strongly agree or agree with the statement compared to only 47% in the booster-hesitant group (Figure 1 and Figure 2).

An independent-sample *t*-test was run to determine if there were differences in functional and critical literacy (FL and CL) between vaccine hesitant and non-hesitant groups. There was homogeneity of variances, as assessed by the Levene’s test for equality of variances (*p* > 0.05). The FL and CL were higher among the vaccine non-hesitant group as compared to the hesitant group with a statistically significant mean difference of −0.49 and −0.41, respectively (Table 4). A Welch *t*-test was run to determine if there were differences in the integrative or communicative literacy and vaccine confidence index between booster hesitant and non-hesitant participants due to the assumption of homogeneity of variances being violated, as assessed by Levene’s test for equality of variances (*p* < 0.05). There were no outliers in the data. The communicative literacy was higher among vaccine non-hesitant group (M = 3.10, SD = 0.60) than hesitant ones (M = 2.70, SD = 0.66), a statistically significant difference, M = −0.40, 95% CI [−0.45, −0.34], t (2136) = −13.992, *p* < 0.001. The vaccine confidence was higher among vaccine non-hesitant group (M = 2.43, SD = 1.08) than hesitant ones (M = 1.20, SD = 0.66), a statistically significant difference, M = −1.23, 95% CI [−1.32, −1.17], t (2136) = −32.960, *p* < 0.001.

As indicated in Table 5, the booster-hesitant group with “a lot” of trust in COVID-19 vaccine information had significantly low mean scores of functional, communicative, and critical vaccine literacies compared to their non-hesitant counterparts.

## 4. Discussion

The main objectives of this study were to measure and identify factors associated with COVID-19 vaccine booster hesitancy and assess the roles of vaccine literacy (VL) and vaccine confidence (VC) in American adults’ willingness to take vaccine boosters. The study was conducted before the approval of COVID-19 vaccine boosters on 23 September 2021, by the CDC’s Advisory Committee on Immunization Practices. With the arrival of new variants and the potential waning of vaccine protection, it appears inevitable that boosters will play an important role in ending the COVID-19 pandemic [7,18,23,39].

The first significant finding in our data relates to vaccination uptake in the general population. A total of 2138 adult Americans aged 18 and over participated in this study. Sixty-eight percent (68%) of participants reported completing or initiating vaccination against COVID-19, and the remaining thirty-two percent (32%) were unvaccinated. Over fifty-seven percent (57%) of the participants in this study reported being fully vaccinated against COVID-19, which is similar to the COVID-19 vaccine acceptance rate (56.9%) found in another study [27]. Furthermore, the percentage of vaccinated individuals in the current national study corresponds to the percentage of fully vaccinated individuals in the U.S. population (56%) as of 5 October 2021 [40]. These data indicate that there is a significant level of under-vaccination in the American adult population. These vaccination rates are significantly less than the estimated herd immunity threshold (60–80%) [41,42,43]. The herd immunity threshold is expected to be higher for the delta variant [44].

The second significant finding relates to self-reported willingness to accept a booster dose in the future once they are recommended. Among participants who were fully vaccinated or have received the first dose, an overwhelming majority (79.1%) would take a booster dose if recommended. In contrast, nearly half of the unvaccinated participants (46.3%) reported they would not take the booster dose. This suggests that acceptance of primary vaccination is strongly associated with willingness to accept a booster if recommended. Furthermore, a quarter of unvaccinated participants (28.6%) and thirteen percent of vaccinated participants (13%) indicate that they are unsure whether they would accept a booster dose. This suggests there are many individuals who have no fixed opinion on booster shots yet and may be persuadable. The additional fact that a quarter (25.1%) of the unvaccinated group was willing to take the booster dose demonstrates the dynamic nature of vaccine hesitancy and the potential to change the minds of a proportion of the vaccine hesitant group. In a previous cohort study of individuals who were hesitant to the COVID-19 vaccine in late 2020, 32% reported receiving at least one dose, and another 37% would likely be vaccinated when re-assessed in early 2021 [45]. Perhaps, with a targeted vaccination campaign focused on those who are persuadable within hesitant groups, vaccination uptake (and eventual booster uptake) could be increased.

### 4.1. Correlates of Booster Hesitancy

The data demonstrated various socioeconomic and demographic factors that are significantly associated with vaccine booster hesitancy. Political affiliation was strongly associated with booster hesitancy; being a registered Democrat was associated with significantly higher booster acceptance than being an Independent or a Republican. This finding was consistent with a study by Callaghan and colleagues, which found significant vaccine refusal among individuals who intended to vote for President Trump in 2020 and were also identified as conservatives [46]. Next, in a bivariate analysis, education level appeared to be highly associated with vaccine booster hesitancy. In particular, those with a college education and graduate degrees were significantly more likely to accept booster doses than those with only a high school education. These findings suggest that educational level and political affiliation are strongly associated with vaccine hesitancy, which may affect the uptake of vaccine boosters and should be considered focal points of any interventions aimed at persuasion to increase booster uptake.

Some findings in the data deserve further consideration and perhaps further study. With regards to gender, females were significantly more booster hesitant than males in the bivariate analysis. This is a surprising finding. A possible explanation may be found in persistent false messaging on social media that COVID-19 vaccines may cause infertility in females or birth defects [47,48]. Another surprising finding is that the study did not find a statistically significant difference in vaccine booster hesitancy among different racial or ethnic groups, which is contrary to our previous study (unpublished) and other studies [46,49], which showed a significant level of COVID-19 vaccine hesitancy among African Americans and Hispanics. This suggests the factors driving vaccine booster hesitancy might differ from hesitancy toward the initial COVID-19 vaccine. Yet another unexpected finding relates to religious affiliation. Some studies have found that those with particular religious leanings are more likely to be COVID-19 vaccine hesitant; consider the Callaghan study that found that those with high levels of religiosity are more hesitant [46]. The results of bivariate analysis in the present study demonstrate that religiously unaffiliated individuals are much more likely to be booster hesitant, while those who are religious (Roman Catholics in particular) are less likely to be booster hesitant. It is unclear what would drive this difference, which may warrant further exploration with expanded studies. It does suggest that religious objections to COVID-19 are not a significant driver of persistent vaccine hesitancy.

### 4.2. Vaccine Literacy and Vaccine Confidence Index

In order to assess the potential underlying causes of vaccine acceptance or hesitancy among study participants, we utilized a validated instrument to determine vaccine literacy (VL) and vaccine confidence, which are predictors of vaccine acceptance according to previous reports [30]. Vaccine literacy is a person’s ability to collect and understand reliable information about immunizations and use the acquired knowledge to make informed decisions to benefit their health [31]. The VL tool is comprised of three components: functional, interactive/communicative, and critical vaccine literacy. Functional literacy refers to competence in reading and writing; interactive/communicative and critical literacy involves advanced skills that allow individuals to derive meaning from information to enable them to make decisions within the context of their own lives [33]. In our study, the mean VL scores for functional, interactive (communicative), and critical literacy were significantly higher in the group that would accept boosters than in the group hesitant to boosters. This mirrors findings from other studies. One study used similar tools to measure the association between VL and vaccine hesitancy; it found an association between the interactive–critical components of VL and vaccine hesitancy, wherein those with higher measures of interactive–critical VL are more accepting of vaccines [50]. Another cross-sectional study carried out in China reported that parents with higher VL scores were more likely to trust vaccines [51]. These findings provide further indications that there appears to be a significant association between vaccine literacy level and vaccine booster hesitancy.

Vaccine confidence encompasses both trust in the safety and efficacy of the vaccine itself, along with trust in the healthcare system that administers it [52]. In our data, the average vaccine confidence index (VCI) was significantly higher in the group accepting of boosters than in the group that was booster hesitant. This finding is consistent with a study that measured influenza vaccine confidence among nursing home residents, which concluded that a higher VCI was a significant predictor of vaccine uptake [53].

In addition, our data indicated a significant difference in the level of trust in COVID-19 vaccine information between those who are accepting of vaccine boosters and those who are booster hesitant. Trust is thought to be a significant factor in vaccination uptake [54]. The lack of trust in vaccine information among those who are vaccine hesitant is partly due to an opposing plague, which the WHO termed an “infodemic”—a plague that spreads and supplies the public with “fake news,” misinformation, and unfounded scientific claims [55]. According to one study, individuals susceptible to this infodemic are linked with an unwillingness to be vaccinated against COVID-19, along with a decreased tendency to advocate for high-risk and vulnerable individuals to receive the vaccine [56]. Currently, the vaccine booster is approved for use in such groups in addition to individuals 65 years and older. Therefore, government agencies or healthcare providers should not assume that the public trusts the information provided to them.

Upon exploring the trust dimension further, results of our subgroup analysis indicated that the booster-hesitant group had significantly low mean scores of functional, communicative, and critical vaccine literacies. In addition, the level of educational attainment was lower among hesitant individuals compared to their non-hesitant counterparts despite “a lot” of trust. This demonstrates that vaccine hesitancy is not only a function of trust but also attributed to one’s perception of low risk for the disease or carefree attitudes. This lends support to developing educational and m-health interventions based on fourth-generation behavioral theories [57]. These interventions may include simple yet interactive messaging, success stories, and testimonials from the community leaders [57]. To summarize, a successful vaccination campaign aiming to address and improve individuals’ ability to appraise information and interventions to promote vaccine acceptance behavior will be critical.

### 4.3. Willingness to Vaccinate Children

Another important issue in the response to the pandemic is the vaccination of children [41,58]. Even though children have a lower risk of complications and disease burden than adults [59], millions of cases and hundreds of COVID-19-related deaths in this population have been reported [58]. Furthermore, children play a substantial role in the continued transmission of the virus within the population [8,16]. Our study demonstrates the existence of significant hesitancy among parents to have their children vaccinated against COVID-19. The survey asked participants whether they would have their children vaccinated against COVID-19 once the vaccine is available and recommended for their child. Among those parents who are vaccinated against COVID-19, about one-fifth (20.1%) indicated that they would not have their children vaccinated, while about fourteen percent (14.5%) were unsure. Among parents who are unvaccinated against COVID-19, about sixty percent (61.4%) indicated that they would not have their children vaccinated, while one-fifth (20.4%) were unsure. These data reflect high and concerning levels of COVID-19 vaccine hesitancy related to the vaccination of children, even among those parents who are vaccinated themselves.

Our data are reflective of the findings of other studies that indicated that parents are unwilling to have their children unvaccinated. Administration of the COVID-19 vaccine has been authorized for the pediatric age group 12–15 years old since May 2021. As of 10 October 2021, only 39% of this group has been vaccinated [40]. According to a report from Kaiser Family Foundation (KFF) COVID-19 Vaccine Monitor, 48% of parents of age 12–17 reported that their child received at least the first dose, and 34% of parents of children aged 5–11 reported that they would vaccinate their child as soon as the vaccine is available for the age group [60]. A study conducted in the U.K. reported that parents’ and guardians’ hesitancy to vaccinate children against COVID-19 when the option is available is mainly motivated by a lower risk of complications of the virus in children [61].

It is important to point out that in our study, 14.5% of vaccinated parents and 20.4% of unvaccinated parents remained unsure about vaccinating their children, which means these parents are potentially persuadable and had not yet developed a firm opinion. Every effort must be made to address the uncertainty experienced by these parents. There is evidence that healthcare professionals can play an essential role in shaping these attitudes by building trust, candidly addressing parents’ concerns regarding the risks and benefits of COVID-19 and its vaccine, and providing parent-specific education [54]. This scenario has the potential to persuade parents to get their children vaccinated and be part of the solution to ending the pandemic.

These data also raise the question of vaccine mandates for children, as is the case with other mandated vaccines linked to school attendance [62]. In addition to the direct health impact COVID-19 may have on children, they also experience significant setbacks to their wellbeing from COVID-19 impact on schools, their social circumstances, and on how society functions. If we have a safe and effective vaccine that could protect children and allow them to go back to school, see their friends, and have a positive social environment once again, it becomes important to use the vaccine to achieve these goals. Given the high levels of hesitancy to have children vaccinated, these considerations provide a potential justification for instituting vaccine mandates for children once COVID-19 vaccines are demonstrated to be safe and effective, licensed, and widely available for children.

### 4.4. Strength and Limitations

This study provides significant insight into factors associated with COVID-19 vaccine booster hesitancy, as well as vaccine hesitancy that may affect vaccine uptake among children. A notable strength of the study is that the sample is nationally representative. Furthermore, the percentage of fully vaccinated individuals in the study (57%) corresponds to the percentage of fully vaccinated individuals in the U.S. population (56%) as of 5 October 2021 [39]. To our knowledge, this is the first study to explore the association between vaccine confidence, vaccine literacy, and booster dose acceptability. The study has several limitations. First, this study arises from a cross-sectional design where data reflects a snapshot of willingness to take the booster vaccine when, in reality, individual attitudes are dynamic and evolving. Second, as is the case for all cross-sectional studies, causality cannot be inferred from this design. Third, though our findings share similar outcomes with previous non-COVID-19 vaccine studies, we cannot confidently confirm our results’ generalizability due to the unique context created by the pandemic. Fourth, web-based surveys are easily susceptible to the effects of self-selection bias—a bias that comes from a non-representative sample restricted to internet users—and often face low response rates. Finally, our study is susceptible to self-reporting, social desirability, and language biases.

This study did not attempt to seek an objective correlation between VCI and VL. However, measuring and understanding their correlation and effects on each other, in a similar setting and context, promise to provide invaluable information in understanding and mitigating vaccine hesitancy. Furthermore, as is the case with vaccine hesitancy, VL and VC are dynamic and context-dependent. Hence, measuring and tracking them over a period of time is essential and represents avenues for further study.

## 5. Conclusions

This article presents the results of a cross-sectional study involving a representative sample of the American population in terms of geography, gender, race, and ethnicity. The data present important insights related to COVID-19-vaccine booster hesitancy, potential factors related to booster hesitancy, and future COVID-19 vaccination uptake. A large majority of those who have been vaccinated against COVID-19 with an initial series report the intention to receive booster shots when boosters become necessary. This is good news, given the fact that booster shots will inevitably play an important role in our response to COVID-19. Of those who were unvaccinated, a significant proportion indicated that they would receive a booster shot or that they were unsure about booster shots, which may indicate that these persons are persuadable. This highlights the need for data on factors that relate to COVID-19 vaccine hesitancy and consideration of the ways to respond to the specific needs of such persons to increase the vaccination uptake. This study also demonstrated that education and vaccine literacy play a significant role in COVID-19 vaccine booster uptake intention. Thought must be given to communicating vaccine science in ways that are accessible to those with lower levels of education or lower levels of vaccine literacy to increase the vaccination uptake. Furthermore, acceptance of vaccine boosters was significantly associated with political affiliation, which underlines the way in which the issue of COVID-19 vaccinations has become politicized. It is an urgent priority to find ways to change this; COVID-19 vaccination is not and should not be a partisan political issue but should be something society can coalesce around. Ways should be sought to remove the politicization around vaccines and to communicate in ways that can draw in adherents of different political persuasions and views. These data and conclusions are important in consideration of future policies and public health interventions aimed at increasing the COVID-19 vaccination uptake and vaccine uptake more generally.

## Figures and Tables

**Table 1:** Quota constraints to recruit nationally representative sample.

Demography	Characteristics	Study Sample (%)	Census Distribution *, Population Parameters (%)
Gender	Female	49.7	50.8
	Male	47.6	49.2
Ethnicity	Non-Hispanic White	61.9	60.1
	Non-Hispanic Black	12.3	13.4
	Hispanic	17.4	18.5
	Asian	5.3	5.9
	Others	3.1	2.1
Region	Midwest	21.33	20.8
	Northeast	18.02	17.1
	South	37.27	38.3
	West	23.38	23.9

* https://www.census.gov/quickfacts/fact/table/US/PST045219 (accessed on 13 November 2021).

**Table 2:** Intention of taking booster dose by sample characteristics (*n* = 2138).

Variable Name	Categories	Intention to Take Booster Dose		*p* Value	Statistics	ES
		Yes (*n* = 1322)	No (*n* = 816)			
Vaccinated status	Yes	1151 (87.1)	305 (37.4)	<0.001	573.43	0.518
	No	171 (12.9)	511 (62.6)			
Intention to have children vaccinated *	Yes	406(72.5)	59 (13.9)	<0.001	332.277	0.581
	No	154 (27.5)	365 (86.1)			
Age (Mean ± SD)	-	47.62 ± 19.3	42.47 ± 17.7			
Gender	Male	680 (51.4)	338 (41.4)	<0.001	29.391	0.117
	Female	611 (46.2)	452 (55.4)	<0.001		
	Other	31 (2.3)	21 (2.6)	0.2		
Race/ethnicity	White	842 (63.7)	513 (62.9)	0.7	5.677	0.052
	African American	156 (11.8)	121 (14.8)	0.05		
	Hispanic	208 (15.7)	125 (15.3)	0.8		
	Other (including multiracial groups)	116 (8.8)	57 (7.0)	0.1		
Marital status	Divorced/Separated/Widowed	283(21.4)	189(23.2)	0.3	51.167	0.155
	Married	659 (49.8)	286 (35.0)	<0.001		
	Single, never married	380 (28.7)	341(41.8)	<0.001		
Education	High school diploma or GED	266 (20.1)	249 (30.5)	<0.001	108.969	0.226
	4-year college degree	345 (26.1)	137 (16.8)	<0.001		
	Graduate level degree	279 (21.1)	77 (9.4)	<0.001		
	Some college	382 (28.9)	284 (34.8)	<0.001		
	Some high school	38 (2.9)	55 (6.7)	<0.001		
	Other	12 (0.9)	14 (1.7)	0.09		
Health insurance	Yes	1214 (93.0)	665 (83.6)	<0.001	50. 668	0.154
	No	92 (7.0)	130 (16.4)			
Friends/Family tested positive for COVID-19	Yes	674 (51.5)	350 (43.9)	0.001	16.797	0.089
	No	635 (48.5)	448 (56.1)			
Living with vulnerable/immunocompromised person	Yes	496 (38.2)	196 (24.9)	<0.001	45.802	0.146
	No	803 (61.8)	591(75.1)			
Pre-existing conditions	Yes	647 (50.0)	298 (38.0)	<0.001	34.162	0.126
	No	648 (50.0)	487 (62.0)			
Region	Midwest	288 (21.8)	193 (23.7)	0.3	21.304	0.100
	Northeast	280 (21.2)	124 (15.2)	<0.001		
	South	440 (33.3)	334 (40.9)	<0.001		
	West	314 (23.8)	165 (20.2)	0.06		
Political affiliation	Democrat	627 (47.4)	203 (24.9)	<0.001	110.494	0.227
	Republican	306 (23.1)	267 (32.7)	<0.001		
	Independent	317 (24.0)	269 (33.0)	<0.001		
	Others	14 (1.1)	11(1.3)	0.5		
Religion	Roman Catholic	344 (26.0)	141 (17.3)	<0.001	28.179	0.115
	Protestant	297 (22.5)	181 (22.2)	0.8		
	Religiously unaffiliated	362 (27.4)	290 (35.5)	<0.001		
	Others	319 (24.1)	204 (25.0)	0.6		

Note: For this analysis, negative and “not sure” responses were combined as “no” category. *p* values are Bonferroni corrected for multiple comparisons; ES: Effect size; SD: Standard deviation. Religiously unaffiliated group includes Agnostic, Atheist, and those with no particular religion. Others include Buddhist, Hindu, Jewish, Mormon, Muslim, Orthodox such as Greek or Russian Orthodox. * Eligible parents = 984 participants.

**Table 3:** Differences in the trust in the COVID-19 information among groups (*n* = 2138).

Variable Name	Categories	Intention to Take Booster Dose		*p* Value	Chi-Square Statistics	ES
		Yes (*n* = 1322)	No (*n* = 816)			
How much trust in COVID-19 vaccine information, n (%)	Not at all	41 (3.1)	197 (24.1)	<0.001	508.481	0.488
	Very little	162 (12.3)	254 (31.1)	<0.001		
	Somewhat	517 (39.1)	292 (35.8)	0.13		
	A lot	602 (45.5)	73 (8.9)	<0.001		

ES: Effect size.

**Table 4:** Vaccine literacy and vaccine confidence among booster dose hesitant and booster dose non-hesitant groups (*n* = 2138).

Variable Name	Intention to Take Booster Dose		*p* Value
	Yes (*n* = 1322)	No (*n* = 816)	
Functional literacy	3.10 ± 0.75	2.61 ± 0.74	<0.001
Integrative or communicative literacy	3.10 ± 0.60	2.70 ± 0.66	<0.001
Critical literacy	3.21 ± 0.68	2.80 ± 0.74	<0.001
Vaccine confidence index	2.43 ± 1.08	1.20 ± 0.66	<0.001

Note: All measures are represented as mean ± standard deviation unless stated otherwise.

**Table 5:** Comparing vaccine literacy and educational attainment among hesitant and non-hesitant groups, who have had “a lot” of trust in COVID-19 information.

Trust “A Lot” in COVID-19 Vaccine Information	Booster Hesitant 73 (8.9)	Booster Non-Hesitant 602 (45.5)	*p* Values	95% CI of the Mean Difference
Vaccine literacy (M ± SD)				
Functional literacy	2.84 ± 0.82	3.20 ± 0.75	<0.001	−0.55, −0.17
Communicative literacy	3.00 ± 0.70	3.40 ± 0.54	0.001	−0.041, −0.15
Critical literacy	3.10 ± 0.76	3.43 ± 0.63	0.003	−0.45, −0.13
Education attainment, n (%)				
High school diploma or GED	4 (5.5)	7 (1.2)	<0.001	
4-year college degree	11 (15.1)	159 (26.4)		
Graduate level degree	12 (16.4)	169 (28.1)		
Some college	17 (23.3)	90 (15.0)		
Some high school	27 (37.0)	174 (28.9)		

## Data Availability

The data presented in this study are available on request from the corresponding author. The data are not publicly available due to ethical reasons.
